# Cardiovascular risk profiles and 20-year mortality in older people: gender differences in the Pro.V.A. study

**DOI:** 10.1007/s10433-021-00620-y

**Published:** 2021-04-12

**Authors:** Caterina Trevisan, Giulia Capodaglio, Eliana Ferroni, Ugo Fedeli, Marianna Noale, Giovannella Baggio, Enzo Manzato, Stefania Maggi, Maria Chiara Corti, Giuseppe Sergi

**Affiliations:** 1 Department of Medicine (DIMED), Geriatric Division, University of Padova, Padua, Italy; 2 Epidemiological System of the Veneto Region, Padova, Italy; 3 National Research Council, Neuroscience Institute, Padova, Italy; 4 Italian Center for the Studies on Gender Health and Medicine, Padova, Italy; 5 AGENAS, National Agency for Regional Health Services, National Outcome Program, Rome, Italy

**Keywords:** Cardiovascular risk factors, Mortality, Gender differences, Older age

## Abstract

The age- and gender-related cardio-metabolic changes may limit the applicability of guidelines for the prevention of cardiovascular diseases (CVD) in older people. We investigated the association of cardiovascular risk profile with 20-year all-cause and CVD-mortality in older adults, focusing on age- and gender-specific differences. This prospective study involved 2895 community-dwelling individuals aged ≥65 years who participated in the Pro.V.A study. The sum of achieved target levels (smoking, diet, physical activity, body weight, blood pressure, lipids, and diabetes) recommended by the European Society of Cardiology 2016 guidelines was assessed in each participant. From this sum, cardiovascular risk profile was categorised as very high (0–2), high (3), medium (4), low (5), and very low (6–7 target levels achieved). All-cause and CV mortality data over 20 years were obtained from health registers. At Cox regression, lower cardiovascular risk profile was associated with reduced 20-year all-cause mortality in both genders, with stronger results for women (HR = 0.42 [95%CI:0.25–0.69] and HR = 0.61 [95%CI:0.42–0.89] for very low vs. very high cardiovascular risk profile in women and men, respectively). This trend was more marked for CVD mortality. Lower cardiovascular risk profile was associated with reduced all-cause and CVD mortality only in men < 75 years, while the associations persisted in the oldest old women. A lower cardiovascular risk profile, as defined by current guidelines, may reduce all-cause and CVD mortality in older people, with stronger and longer benefits in women. These findings suggest that personalised and life-course approaches considering gender and age differences may improve the delivery of preventive actions in older people.

**Supplementary Information:**

The online version contains supplementary material available at 10.1007/s10433-021-00620-y.

## Introduction

Cardiovascular diseases (CVD) are the most frequent pathologies in adult and older age, and their prevalence is set to increase further due to longer lifespans in high-income countries (Christensen et al. 2009; Global status report on noncommunicable diseases 2010 2011). The guidelines for CVD prevention of the European Society of Cardiology (ESC) define seven major cardiovascular (CV) risk factors, namely: smoking, diet, physical activity, body weight, blood pressure, lipids, and diabetes (Piepoli et al. 2016). For each factor, target levels associated with a lower CV risk have been proposed. Although these recommendations are supported by a consolidated literature, the authors of such guidelines acknowledge that there are gaps in our knowledge, especially concerning older people and the female gender (Piepoli et al. 2016).

Indeed, the target ranges for CV risk factors were generally determined from studies in which older people and women were underrepresented (Trevisan et al. 2017), yet in light of age-related changes in the cardio-metabolic system, the same cutoffs might not be applicable to older individuals (Ruijter et al. 2009; Störk et al. 2006). Moreover, the extent to which these risk factors may impact on survival can vary even between the youngest and the oldest old, suggesting that more relaxed cutoffs be adopted for factors, such as body weight (Winter et al. 2014), blood pressure or glycaemia in later life (Piepoli et al. 2016).

Gender may be an additional factor influencing the association between CV risk and survival over time (Trevisan et al. 2017; Maas et al. 2011). Indeed, women after menopause become progressively more vulnerable to CVD, thus they are generally older than men at CVD onset and have more comorbidities and specific risk factor patterns (Trevisan et al. 2017; Maas et al. 2011).

Several cohort studies provided evidence for the influence of risk behaviours, alone or in combination, on survival also in older age (Knoops et al. 2004; Kvaavik 2010; Khaw et al. 2008; Haveman-Nies 2003; Ford et al. 2011; Petersen et al. 2015; Odegaard et al. 2011). However, these studies have limited comparability because of the different cutoffs they use. Moreover, their exclusive focus on lifestyle does not consider age-related cardiometabolic changes, which may further affect older people’s cardiovascular risk profiles. The seven factors mentioned in the ESC guidelines reflect this view and highlight the need for comprehensive assessment of both lifestyle and CV endpoints, such as blood pressure, lipids, and glycaemia levels. However, to date no studies have looked at how CV risk profiles determined according to ESC guidelines impact on survival in older people and the additional potential influence of advanced age and gender.

We thus investigated whether a low CV risk profile, defined as the achievement of validated targets, was associated with lower all-cause and CV mortality in older people over a 20-year follow-up, and if this relationship differed according to gender and age. Our hypotheses were that a low-risk CV profile is associated with longer survival also in older age, and that this relationship has gender-specific features.

## Methods

### Study population

Data come from the *Progetto Veneto Anziani* (Pro.V.A.), a cohort study carried out in two towns in north-eastern Italy. The baseline assessment was performed in 1995–1997 and involved 3099 individuals aged ≥ 65 years, randomly selected according to a multistage stratified method (overall response rate: 77%M, 64%F) (Corti et al. 2002). Information on participants’ vital status up to 2017 was obtained from administrative data.

For this study, of the initial 3099 participants, we excluded 118 institutionalised individuals, 85 with missing data on CV risk factors and one individual whose vital status data were not retrieved from the registers. The final analytical sample was 2895 individuals (for the comparison between participants included and excluded from the study please see Online Appendix 1).

The study protocol was approved by the ethics committees of the University of Padova, Local Health Units15 and 18 of the Veneto Region, and the Province of Padova, and all participants gave their written informed consent.

The study results are reported according to the STROBE recommendations (Elm et al. 2007).

### Participant assessment

Baseline assessments of the study participants were carried out by trained physicians and nurses in clinics or, when unable to attend a clinic, at home. The assessments were made through face-to-face interviews, examination of medical records, standardised questionnaires, and physical examinations.

#### CV risk profile

At baseline, we evaluated achievement of the target levels recommended by the ESC 2016 guidelines (Piepoli et al. 2016) for the following factors:

1. Smoking: taking absence of exposure to tobacco in any form (i.e. never/former *vs* current smokers) as healthy.
2. Diet: a composite measure of dietary habits was obtained from self-reports and food-frequency questionnaires on the following: fat intake (self-report on adherence to a low-fat diet); alcohol consumption (healthy if less than 20 g/day[M] or 10 g/day[F]); consumption of fruit (healthy if ≥ 200 g/day) and vegetables (healthy if ≥ 200 g/day); omega-3/omega-6 intake (healthy with fish intake ≥ 1–2 times/week or unsalted nut intake ≥ 30 gr/day). A healthy diet was defined as adherence to at least three of these five healthy dietary habits.
3. Physical activity level: defined as healthy if participants reported ≥ 150 min/week of moderate aerobic exercise (e.g. walking briskly, gardening, ballroom dancing) or ≥ 75 min/week of vigorous aerobic exercise (e.g. race walking, jogging), exclusively or in combination.
4. Body weight: defined as healthy with a BMI of 20–25 kg/m^2^ and a waist circumference < 94 cm[M] or < 80 cm[F]. Body weight and height were measured with individuals wearing light indoor clothing and without shoes. BMI was computed as the ratio between a person’s body weight and height squared. Waist circumference was measured midway between the lowest rib and the iliac crest with participants standing.
5. Blood pressure: defined as healthy if systolic blood pressure was < 140 mmHg and diastolic blood pressure was < 90 mmHg. Both blood pressure readings were taken by trained nurses with patients in the clinostatic position. Three readings were taken at 30-s interval on both arms using a mercury sphygmomanometer (Erkameter 300), and blood pressure was determined as the mean of these three values.
6. Lipids: LDL-cholesterol levels were defined as healthy if < 1.8 mmol/l in very high risk individuals, < 2.6 mmol/l in high risk individuals, and < 3.0 mmol/l in low-to-moderate risk individuals. Risk categories were calculated in accordance with ESC 2016 guidelines (Piepoli et al. 2016) and the Systematic COronary Risk Evaluation-Older Persons (SCORE-OP) (Cooney et al. 2016).
7. Diabetes: healthy glycaemic control was defined as the absence of diabetes or, in diabetic people, a glycated haemoglobin (HbA1c) < 7%. Where no HbA1c data (*n* = 206) were available, a fasting glycaemia ≤ 130 mg/dl was considered a proxy for healthy glycaemic control (American Diabetes Association 2018,2017).

We obtained an overall score for each study participant from the sum of the risk factor variables within target levels, ranging from 0 (no target level achieved: worst CV risk profile) to 7 (all target levels achieved: best CV risk profile). Based on the overall score distribution in our sample, the CV risk profile was categorised as: very high (0–2 target levels achieved), high (3 target levels achieved), medium (4 target levels achieved), low (5 target levels achieved), and very low (6–7 target levels achieved).

#### Sociodemographic, clinical, and biochemical data

For each participant, we collected data on basic sociodemographic information, educational level, monthly income, and on the presence of: hypertension; chronic obstructive pulmonary disease (COPD); cancer; osteoarticular diseases; and CVD, defined as the presence of at least one of the following: congestive heart failure, angina requiring a stent, angioplasty or hospitalisation, myocardial infarction and stroke. We also recorded the use of lipid lowering drugs (LLD). Moreover, to integrate the information on CV risk profile, we collected information on the number of years since former smokers quit smoking, and since diabetes diagnosis, for those who were affected by such disease.

Cognitive impairment was defined as a Mini-Mental State Examination (MMSE) < 24, and the presence of depressive symptoms as a 30-item Geriatric Depression Scale (GDS) > 10. Biochemical analyses were performed to assess LDL-cholesterol, HbA1c, fasting glycaemia, serum creatinine (used to compute the glomerular filtration rate [GFR, ml/min]), fibrinogen levels, and the erythrocyte sedimentation rate (ESR). Details on these assessments can be found in Online Appendix 2.

#### Vital status

The dates and causes of death of participants who had died by the 31 December 2017 were obtained from regional health registers.

### Statistical analysis

The baseline participants’ characteristics were compared by CV risk profile using the Student’s t-test for continuous variables and the Chi-squared test for categorical variables. The rate of missing MMSE values was > 5% (*n* = 349), so that we performed a single imputation using the expectation maximisation algorithm.

Age-standardised mortality rates of participants categorised by CV risk profiles were computed using the direct standardisation method and the population of the Veneto Region on 1st January 2007. The associations between CV risk profile and mortality in older men and women were determined by Cox regression, considering the worse CV risk profile category as reference and age as the time scale. The strength of these associations was estimated through hazard ratios (HR) and 95% confidence intervals (95%CI), adjusted for potential confounders. Analyses were performed stratified by gender and then also by age groups (65–74, 75–84, ≥ 85). As sensitivity analyses, we tested the above association in participants free from CVD at baseline (*n* = 2256), and we explored the impact of single CV risk factors on mortality. Interactions between age and gender in influencing CV risk profile and mortality were tested by including the multiplicative interaction term in a cumulative logit model, for the first outcome (i.e. CV risk profile), and in the fully-adjusted Cox model, for the second outcome (i.e. mortality). All statistical tests were two-tailed, and statistical significance was assumed for a p-value < 0.05. The analyses were performed using SAS, V.9.4 (SAS Institute, Cary, NC) and SPSS 23.0 for Windows (Armonk, NY: IBM Corp).

## Results

**Fig. 1:**
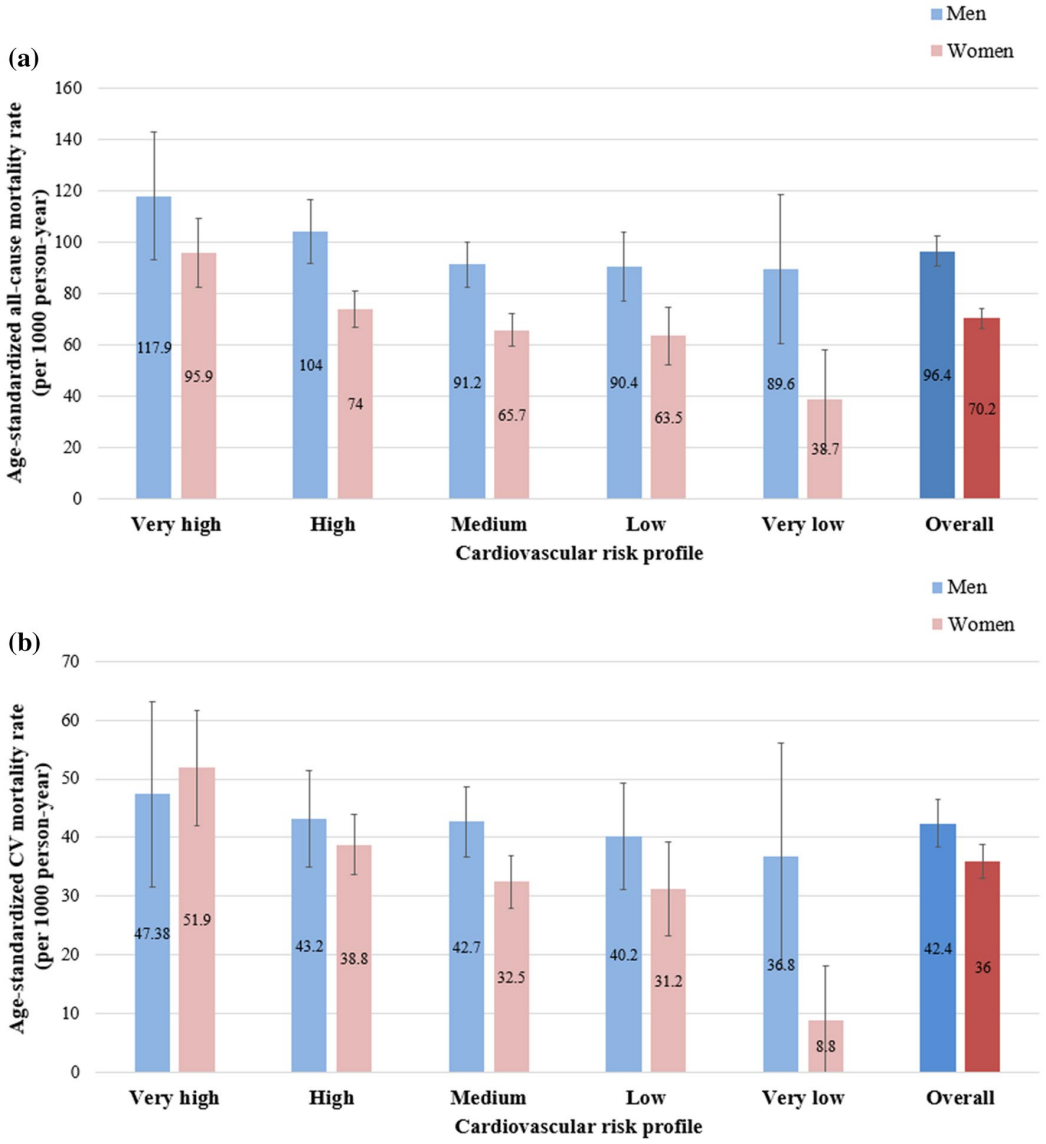
Age-standardised all-cause **a** and cardiovascular **b** mortality rate by gender and by cardiovascular risk profile. *Notes.* Bars represent 95% confidence interval. Mortality rates were computed through direct standardisation, using Veneto Region at 1st January 2007 as the standard population

The characteristics of men and women by CV risk profile are reported in Tables 1 and 2, respectively. Significant differences emerged in the percentages of men (*n* = 1187) and women (*n* = 1708) in the very low (4.3%[M] vs. 2%[F], *p* < 0.001), low (17.7%[M] vs. 14.6%[F], *p* = 0.03), and very high (9.6%[M] vs. 12.5%[F], *p* = 0.02) CV risk categories. Comparing the genders for the prevalence of CV risk factors within target levels (Figure 1), we found that women were less likely to be current smokers and more likely to have healthy dietary habits, while men were more likely to have a healthy body weight, high physical activity levels (*p* < 0.001 for all), and better glycaemic control (*p* = 0.03). Considering former smokers, the median number of years since smoking cessation was 18 (IQR: 10–29), and it was slightly longer for men than women (median 18 vs. 15 years). Conversely, no sex-specific differences were observed for the median number of years since diabetes diagnosis, which was equal to 9.8 (IQR: 3.3–17.2). A significant interaction of age and gender was found with respect to the CV risk profile (*p* < 0.001), i.e. participants with the healthiest CV profiles were the oldest of the men, but the youngest of the women. As expected, we found the higher the CV risk profile, the higher the prevalence of CVD at baseline in both genders, with more marked results for men.

**Table 1:** Baseline characteristics of the 1187 men stratified by cardiovascular risk profile

Baseline Characteristics	All (*n* = 1187)	Cardiovascular risk profile				
		Very low (*n* = 51)	Low (*n* = 210)	Medium (*n* = 486)	High (*n* = 326)	Very high (*n* = 114)
*Sociodemographic data*						
Age (*y*)	76.2 ± 7.8	76.7 ± 8.6	77.1 ± 8.4	76.7 ± 7.6	75.1 ± 7.3	74.6 ± 7.6**
Education ≥ 5 *y* (%)	66.9	64.7	65.7	69.1	65.6	64
Monthly income > 500€ (%)	51.5	43.1	42.9	54.7	53.7	50.9*
Living alone (%)	8.9	11.8	8.1	8.4	8.6	12.3
*Multidimensional assessment*						
Walking speed (m/s)	0.81 ± 0.22	0.82 ± 0.19	0.81 ± 0.21	0.82 ± 0.22	0.8 ± 0.23	0.78 ± 0.2
MMSE < 24 (%)	35	39.2	36.2	32.7	35.9	38.6
GDS ≥ 11 (%)	27	29.4	28.1	25.1	30.1	22.8
*Biochemical analyses*						
ESR (mm/h)	15.9 ± 17.7	10.9 ± 11.2	17.6 ± 16.7	15.7 ± 19.8	16.2 ± 17.4	15 ± 12.6
Fibrinogen (mg/dl)	339.3 ± 131.1	305.4 ± 63.2	329.9 ± 76.1	341.7 ± 178	345.4 ± 93.2	344.9 ± 73.2
GFR (ml/min/1.73 m^2^)	73.8 ± 19	77.5 ± 18.5	73.3 ± 17.2	73 ± 20	74.8 ± 18.4	73.5 ± 19.3
*Chronic diseases*						
CVD (%)	28.4	9.8	25.2	26.3	32.8	38.6**
COPD (%)	16.1	7.8	18.6	16	15	18.4
Osteoarticular diseases (%)	44.2	58.8	43.8	43.4	43.6	43.9
Cancer (%)	9.2	7.8	8.1	9.1	12	4.4
Use of LLD (%)	2.8	2	3.3	2.9	2.1	3.5
*Cardiovascular healthy factors*						
No current smoking habits (%)	83.9	98	92.4	90.3	81.6	41.2***
Healthy diet (%)	56.9	98	84.8	71	27.3	11.4***
Healthy physical activity (%)	83.4	100	93.3	87	77.3	59.6***
Healthy weight (%)	24.6	86.3	50.5	22.6	9.2	1.8***
BMI (kg/m^2^)	26.8 ± 3.9	23.4 ± 3	25.4 ± 3.7	26.8 ± 3.6	27.5 ± 3.8	28.8 ± 3.9***
Waist (cm)	97.3 ± 10	87.3 ± 8.3	93.4 ± 9.7	97.2 ± 9.5	99.7 ± 9.3	103 ± 9.6***
Healthy blood pressure (%)	31.8	86.3	71	28.8	12.6	3.5***
Healthy LDL-cholesterol (%)	4.9	41.2	8.6	3.5	0.6	0***
Healthy glucose control (%)	93.3	100	99.5	96.7	91.4	69.3***

Continuous variables are expressed as mean ± standard deviation. *Abbreviations:* BMI, Body mass index; MMSE, Mini-Mental State Examination; ESR, erythrocyte sedimentation rate; GFR, glomerular filtration rate; CVD, cardiovascular diseases; COPD, Chronic Obstructive Pulmonary Disease; LLD, lipid lowering drugs. *p* values refer to the comparison between different cardiovascular risk profile groups.

*p < 0.05; **p < 0.01; ***p < 0.001

**Table 2:** Baseline characteristics of the 1708 women stratified by cardiovascular risk profile

Baseline characteristics	All (*n* = 1708)	Cardiovascular risk profile				
		Very low (*n* = 35)	Low (*n* = 250)	Medium (*n* = 684)	High (*n* = 526)	Very high (*n* = 213)
*Sociodemographic data*						
Age (*y*)	75.5 ± 7.4	72.1 ± 7	73.8 ± 6.8	74.5 ± 7	76.6 ± 7.6	78.6 ± 7.3***
Education ≥ 5 *y* (%)	41.6	22 (62.9)	112 (44.8)	303 (44.3)	199 (37.8)	75 (35.2)**
Monthly income > 500€ (%)	30.9	15 (42.9)	62 (24.8)	217 (31.7)	165 (31.4)	69 (32.4)
Living alone (%)	23.8	8 (22.9)	56 (22.4)	184 (26.9)	119 (22.6)	39 (18.3)
*Multidimensional assessment*						
Walking speed (m/s)	0.7 ± 0.21	0.82 ± 0.24	0.74 ± 0.18	0.73 ± 0.2	0.66 ± 0.22	0.59 ± 0.21***
MMSE < 24	43.7	13 (37.1)	97 (38.8)	244 (35.7)	257 (48.9)	135 (63.4)***
GDS ≥ 11	43.2	12 (34.3)	97 (38.8)	289 (42.3)	244 (46.4)	96 (45.1)
*Biochemical analyses*						
ESR (mm/h)	23.9 ± 18.1	17.4 ± 12.5	22.4 ± 17.1	22.6 ± 16.5	25 ± 19.5	28.5 ± 20.5***
Fibrinogen (mg/dl)	353.2 ± 80.8	316.9 ± 79.4	339.2 ± 72.9	349.1 ± 77.5	361.3 ± 84.6	369.8 ± 85.6***
GFR (ml/min/1.73 m^2^)	66.3 ± 18.1	71.3 ± 13.9	67 ± 17.1	66.6 ± 17.1	65.2 ± 17.2	66.8 ± 23.9
*Chronic diseases*						
CVD (%)	17.7	0	10.8	14.6	22.8	25.8***
COPD (%)	5.2	0	3.6	3.5	7.2	8.5**
Osteoarticular diseases (%)	72.2	54.3	68	69.6	77.8	75.1**
Cancer (%)	6.7	5.7	7.2	5.6	8.2	6.1
Use of LLD (%)	4.2	2.9	3.6	6.3	2.9	1.4**
*Cardiovascular healthy factors*						
No current smoking habits (%)	95.8	100	98.4	97.5	95.6	86.9***
Healthy diet (%)	66.4	100	97.2	84.5	45.8	17.4***
Healthy physical activity (%)	65.5	97.1	94	81.1	50	14.6***
Healthy weight (%)	5.3	45.7	9.6	5.4	2.3	0.9***
BMI (kg/m^2^)	28.1 ± 4.9	26.2 ± 4.2	27.5 ± 4.6	28.1 ± 4.7	28.3 ± 5.1	28.6 ± 5.7*
Waist (cm)	95.9 ± 12.4	88.3 ± 14	93.1 ± 11.8	95.6 ± 12.2	97.2 ± 11.9	98.3 ± 13.5***
Healthy blood pressure (%)	32.4	94.3	85.2	31.4	16.2	3.8***
Healthy LDL-cholesterol (%)	5.6	62.9	16	3.4	2.1	0***
Healthy glucose control (%)	91	100	99.6	96.6	88	68.5***

Continuous variables are expressed as mean ± standard deviation. P-values refer to the comparison between different cardiovascular risk profile groups. *Abbreviations:* BMI, Body mass index; MMSE, Mini-Mental State Examination; ESR, erythrocyte sedimentation rate; GFR, glomerular filtration rate; CVD, cardiovascular diseases; COPD, Chronic Obstructive Pulmonary Disease; LLD, lipid lowering drugs.

*p < 0.05; **p < 0.01; ***p < 0.001 for the difference between cardiovascular risk profile categories

**Fig. 2:**
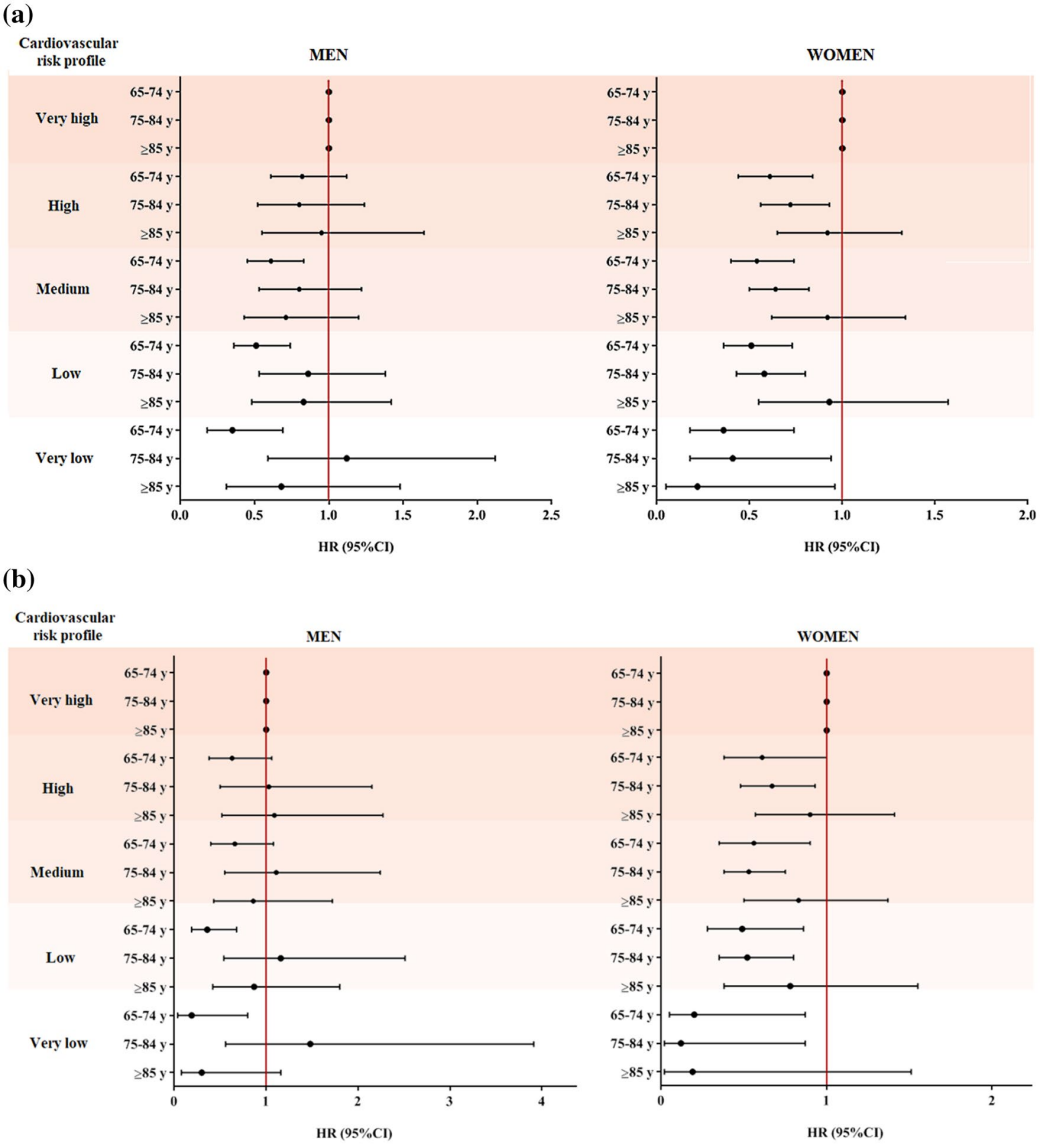
Hazard ratios of all-cause **a** and cardiovascular **b** mortality by gender, age class, and cardiovascular risk profile. *Abbreviations*: HR, Hazard ratio; 95%CI, 95% Confidence interval. *Notes*: Models adjusted for educational level (≥ 5 vs < 5 years), monthly income (< vs. ≥ 500€), living arrangements (living with somebody vs living alone vs living in nursing home), chronic obstructive pulmonary disease, osteoarticular diseases, cancer, cognitive impairment, cardiovascular diseases

Over the 20-year follow-up, 2373 deaths (1051 M, 1322F) were recorded, and almost half of these (1105; 449 M, 656F) were caused by CVD. The age-standardized rates of all-cause and CV mortality in men and women are shown in Fig. 1a (all-cause) and b (CV). Healthier CV profiles resulted in lower mortality rates in both genders, with more marked trends among women, especially for CV mortality. At Cox regression (Table 3), compared with the worst category, all-cause mortality among men reduced in the lower CV risk profile groups, especially at the 20-year follow-up (high profile: HR = 0.83, 95%CI:0.67–1.04; very low profile: HR = 0.61, 95%CI:0.42–0.89). The decreasing trend of all-cause mortality by lower CV risk profile was more marked for women, with around a 60% decrease in all-cause mortality for the category with the healthiest CV profile. As for CV mortality, although there was a decreasing trend in HRs with lower CV risk profiles in both genders, the results were significant only for women. The findings were confirmed among participants free from CVD at baseline (Table 4). When stratifying the results by age class (Fig. 2, Table A1), the lower CV risk profiles of women were associated with reduced all-cause mortality risk in both the youngest and the oldest old, and with lower CV mortality in women up to 84 years of age. Conversely, the lower CV risk profiles of men were associated with reduced all-cause and CV mortality only in the youngest old. No statistically significant multiplicative interactions were found between gender, age, and CV risk profiles with regards to their influence on mortality. Among the single CV risk factors, a high level of physical activity, good glucose control and no current smoking habits reduced mortality risk over 20 years in both genders. Moreover, only among women healthy diet reduced the 10-year and blood pressure the 20-year CV mortality, while low LDL-cholesterol was associated with increased all-cause mortality (Table A2)).

**Table 3:** Cox regression on the associations between cardiovascular risk profile with 10- and 20-year all-cause and cardiovascular mortality in older men and women

Hazard ratios and 95% confidence intervals for mortality				
	All-cause		Cardiovascular	
	Men	Women	Men	Women
Cardiovascular risk profile	10-year follow-up			
Very high	1.00	1.00	1.00	1.00
High	0.85 (0.64–1.13)	0.78 (0.62–0.97)*	0.81 (0.53–1.24)	0.76 (0.56–1.02)
Medium	0.72 (0.55–0.94)*	0.67 (0.54–0.85)***	0.72 (0.48–1.08)	0.56 (0.40–0.76)***
Low	0.68 (0.50–0.93)*	0.58 (0.42–0.78)***	0.65 (0.41–1.03)	0.45 (0.29–0.71)***
Very low	0.71 (0.44–1.14)	0.34 (0.14–0.84)*	0.55 (0.25–1.18)	0.14 (0.02–1.00)*
*p per trend*	0.0069	< 0.001	0.0327	< 0.001
	20-year follow-up			
Very high	1.00	1.00	1.00	1.00
High	0.83 (0.67–1.04)	0.76 (0.64–0.90)**	0.83 (0.58–1.19)	0.71 (0.56–0.90)**
Medium	0.68 (0.55–0.85)***	0.69 (0.58–0.82)***	0.82 (0.59–1.16)	0.62 (0.49–0.78)***
Low	0.67 (0.52–0.85)**	0.65 (0.53–0.81)***	0.75 (0.51–1.09)	0.58 (0.43–0.78)***
Very low	0.61 (0.42–0.89)**	0.42 (0.25–0.69)***	0.55 (0.30–1.03)	0.20 (0.07–0.54)**
*p per trend*	< 0.001	< 0.001	0.0624	< 0.001

Model adjusted for age, educational level (≥ 5 vs. < 5 years), monthly income (< vs. ≥ 500€), living arrangements (living with somebody vs living alone), chronic obstructive pulmonary disease, osteoarticular diseases, cancer, cognitive impairment, cardiovascular diseases.

*p < 0.05; **p < 0.01; ***p < 0.001

**Table 4:** Association between cardiovascular risk profile with 10- and 20-year all-cause and cardiovascular mortality in men and women with no history of cardiovascular diseases at baseline

Hazard ratios and 95% confidence intervals for mortality				
	All-cause		Cardiovascular	
	Men	Women	Men	Women
Cardiovascular risk profile	10-year follow-up			
Very high	1.00	1.00	1.00	1.00
High	0.77 (0.52–1.14)	0.73 (0.56–0.96)*	0.56 (0.31–1.03)	0.62 (0.43–0.89)**
Medium	0.72 (0.49–1.04)	0.64 (0.48–0.83)***	0.63 (0.36–1.09)	0.47 (0.32–0.69)***
Low	0.63 (0.42–0.95)*	0.52 (0.37–0.75)***	0.46 (0.25–0.85)*	0.35 (0.20–0.61)***
Very low	0.62 (0.36–1.08)	0.31 (0.13–0.78)*	0.38 (0.15–0.95)*	0.11 (0.02–0.80)*
*p per trend*	0.03	< 0.001	0.03	< 0.001
	20-year follow-up			
Very high	1.00	1.00	1.00	1.00
High	0.85 (0.63–1.12)	0.73 (0.60–0.90)**	0.70 (0.44–1.14)	0.64 (0.48–0.84)**
Medium	0.73 (0.55–0.96)*	0.66 (0.54–0.80)***	0.88 (0.57–1.37)	0.57 (0.44–0.75)***
Low	0.69 (0.51–0.93)*	0.62 (0.49–0.78)***	0.63 (0.38–1.03)	0.52 (0.38–0.73)***
Very low	0.59 (0.39–0.90)*	0.40 (0.24–0.67)***	0.44 (0.21–0.91)*	0.19 (0.07–0.52)**
*p per trend*	0.002	< 0.001	0.05	< 0.001

Model adjusted for age, educational level (≥ 5 vs. < 5 years), monthly income (< vs. ≥ 500€), living arrangements (living with somebody vs living alone), chronic obstructive pulmonary disease, osteoarticular diseases, cancer, cognitive impairment, cardiovascular diseases.

*p < 0.05; **p < 0.01; ***p < 0.001

## Discussion

Our study shows that achieving healthy target levels for CV risk factors, as proposed by the latest ESC guidelines, is associated with lower 10- and 20-year all-cause and CV mortality rates, even in older people. However, these beneficial effects are subject to gender differences: in older women the association between CV risk profile and mortality seems to be stronger than in men and to persist in more advanced age.

In our cohort, around one in five participants had a low or very low CV risk profile, defined as the achievement of target levels for most of the risk factors evaluated. Interestingly, gender differences already appeared on looking at the mean age of men and women by CV risk level. The age of participants increased with the worsening of CV risk in women, while men exhibited the opposite trend, i.e. the lower CV risk categories were associated with the oldest mean age. Although the physio-pathological changes occurring in women with aging can explain the accumulation of risk factors among the oldest old, our data for men may indicate selective survival of those having overcome the high-risk period of middle-age.

In this regard, it should be considered that, firstly, the assessment of some CV risk factors in older individuals, as indicated in the ESC guidelines, may not always reflect their lifelong CV risk profiles. This issue concerns, for example, tobacco exposure, for which only current smoking is assessed, or dietary style and physical activity level. Indeed, individuals with better CV profiles may have either maintained healthy behaviours over their life course or modified their lifestyle and corrected their CV endpoints as a consequence of treatments or preventive actions. However, it is noteworthy that our main findings were confirmed in participants free from CVD. This result is consistent with the hypothesis that the CV risk profiles we assessed were influenced more by the individuals’ lifestyles and functional homeostasis, than by medical interventions.

Secondly, certain CV health factors seemed to be poorly represented in our participants, with the recommended target levels achieved by only a few individuals. This was especially the case for body weight and LDL cholesterol, confirming that the nutritional thresholds for adults may not be fully applicable to older people (Sergi et al. 2005). In particular, it should be borne in mind that, compared with underweight or obesity, mild-to-moderate overweight in the older population has been associated with reduced mortality (Winter et al. 2014; Sergi et al. 2005).

These issues need to be taken into account when interpreting our results and support the adoption for older individuals of risk stratification approaches that would consider life-course risk exposures and age- and sex-specific at-risk thresholds. Nonetheless, overall, this study suggests that achieving the recommended target levels of a greater number of CV risk factors, as indicated by the current ESC guidelines, may gradually reduce all-cause and CV mortality even in older age.

The extent of this protective effect is in line with previous work that has found 50–60% reductions in all-cause mortality for various combinations of healthy lifestyle factors (Knoops et al. 2004). When considering potential gender differences, we found that this association was stronger in women than in men, and that the benefits of a healthy CV risk factor profile for survival seemed to last longer in women, persisting to over the age of 75 years. These findings corroborate the slight gender differences observed in previous studies (Khaw et al. 2008; Li et al. 2018) and lead us to put forward two possible hypotheses: either the mechanisms through which CV health factors influence mortality differ by gender and by age period, or the mechanisms are the same but operate in gender-specific time windows of susceptibility. Regarding the first hypothesis, the effect of risky behaviours on health-related outcomes involves inflammatory and oxidative pathways, which increase the risk of cardiometabolic dysfunction and worsen related outcomes (Rizzuto and Fratiglioni 2014). Avoiding these risk factors and satisfactorily controlling cardiometabolic dysfunctions have demonstrated similar benefits in men and women (Kvaavik 2010; Petersen et al. 2015; Odegaard et al. 2011) and in older age (Li et al. 2018; Rawshani et al. 2018). Such an effect may benefit CV health and prevent other chronic conditions, such as pulmonary, osteoarticular, neurologic diseases, affecting both all-cause and CV mortality. Concerning CVD, in particular, our results on individuals with no history of CVD at baseline support a possible preventive action of healthier risk profiles also in older age in delaying the development and progression of such pathologies.

These data strengthen our second hypothesis, namely that age and gender differences are not to do with the mechanisms, but with the extent to which CV health factors influence survival: the greater effect of these protective factors in older women could be due to their greater vulnerability to CVD in advanced age, unlike men, whose high-risk period is middle-age (Hippisley-Cox et al. 2010). This phenomenon may be influenced by the lower levels of female hormones after menopause and subsequent metabolic changes, and by the greater prevalence in females of conditions such as osteoarthritis and obesity, which increase CVD risk and worsen prognosis (Trevisan et al. 2017; Wilson et al. 2002; Tankó et al. 2005). Accordingly, CVD onset is almost 10 years later in women than in men, and women are more likely to present a higher number of risk factors and comorbidities at CVD diagnosis (Sharma and Gulati 2013). In contrast to the timing hypothesis for hormone replacement therapy (Clarkson et al. 2013), healthy behaviours and greater disease control seem also to benefit the oldest old, suggesting that women’s vulnerability to such factors extends into advanced age. Finally, it is to be noted that generational changes could have also influenced the age and gender differences in the association of each factor with mortality. In this regard, further research comparing the extent to which single risk factors affected mortality between different generations of older men and women will be of high interest.

In addition to the observational nature of the study, one of the limitations is the simple assessment of the number of healthy CV factors without considering their potentially different weights (Rizzuto and Fratiglioni 2014). We chose this approach since we wanted to be consistent with the guidelines recommendations that did not prioritise the achievement of any specific target level. However, the sensitivity analysis exploring the association between each CV risk factor and mortality provided some information in this regard. Secondly, as mentioned above, our evaluation of CV risk factors could be biased by possible socially- or medically-induced changes in the years before the baseline visit, as well as by risk factors variations during the follow-up. Together with the use of self-reported information, this issue could be a source of misclassification. This may concern factors such as tobacco exposure (since never and former smokers were both considered as non-exposed), dietary style, and physical activity level. However, as regards smoking habits, the median number of years since smoking cessation in the former smokers of our sample makes unlikely a substantial impact of previous tobacco exposure on 20-year mortality (Gallucci et al. 2020). Considering dietary patterns, changes in sensory and masticatory functions (Sergi et al. 2017; Tada and Miura 2014) might have slightly influenced the preferences and consistency of the foods in our aging population, but should not have caused relevant variations in dietary style (Tada and Miura 2014). Furthermore, despite the possibility of residual confounding, the median time since diabetes diagnosis in our sample and the epidemiological data on age at disease onset (Sattar et al. 2019) can rule out that a substantial number of individuals might have experienced changes in glycemic control or incident diabetes over the follow-up. Third, we included only Caucasian older adults living in northern Italy; therefore, our results are likely to be generalised to similar Italian and European populations. Moreover, we did not include institutionalised individuals because of the possible influence of co-existing conditions highly prevalent in such population (e.g. frailty, multimorbidity, and disability) on the association between CV risk profile and mortality. Possible variability linked to geographical or ethnic differences and the nursing home setting should be investigated in future studies. On the other hand, our work is strengthened by using reliable administrative data on mortality derived from regional health registers, as well as the 20-year follow-up period and comprehensive data collection in a large cohort of older adults.

In conclusion, our study shows that healthier CV risk profiles in older people are associated with reduced all-cause and CV mortality, suggesting the potential effectiveness of preventative action even in advanced age. Although our data show that the current guidelines are also applicable to older individuals, differences by gender and class of advanced age highlight the need for a personalised and life course approach to delivering care and preventive interventions to older men and women in various settings.

## Supplementary Information

Below is the link to the electronic supplementary material.

Supplementary file1 (DOCX 61 kb)
