# Supplementary material for: Cardiovascular risk profiles and 20-year mortality in older people: gender differences in the Pro.V.A. study
Source: Eur J Ageing. 2021 Apr 12;19(1):37–47. doi: 10.1007/s10433-021-00620-y (PMC8881539; doi:10.1007/s10433-021-00620-y)
Supplement: Supplementary file 1 — Supplementary file1 (DOCX 61 kb) [file 10433_2021_620_MOESM1_ESM.docx]

**SUPPLEMENTARY MATERIAL**

**Appendix 1. Comparison of baseline characteristics between participants included and excluded from the study**

Compared with participants included in the main analytical sample (n=2895), the 204 excluded individuals were significantly older (84.6±7.4 vs 75.8±7.5 years, p<0.001) and more likely to be women (72.1% vs 59%, p<0.001), and reported a higher prevalence of diabetes (24% vs 16.1%, p=0.003) and cardiovascular diseases (47.5% vs 22.1%, p<0.001). They reported also lesser health risk behaviors (never smokers: 79.4% vs 60.1%, p<0.001; abstemious: 89.2% vs 69.5%, p<0.001), but were more likely to have cognitive impairment (83.8% vs 40.1% had a Mini-Mental State Examination [MMSE] <24, p<0.001). No significant differences were observed in body mass index (BMI), chronic obstructive pulmonary disease diagnosis (COPD) or cancer.

**Appendix 2. Assessments of sociodemographic, clinical and biochemical data**

***Sociodemographic and clinical data.*** In addition to basic sociodemographic information (age, sex and living arrangements), we collected data on each participant’s educational level (categorised as ≤5 or >5 years, 5 years being the duration of primary schooling in Italy) and monthly income (≤ *vs* >€500). Clinical status was assessed from standardised questionnaires, self-reported symptoms, medical and hospital records, blood tests, physical examination and medication being taken. At baseline, we recorded the presence of: hypertension, diagnosed according to validated guidelines for the adult population^1^; chronic obstructive pulmonary disease (COPD); cancer; osteoarticular diseases, defined as the presence of osteoporosis, or a history of hip fracture or osteoarthritis in the hand, knee or hip; and cardiovascular diseases (CVD), defined as the presence of at least one of the following: congestive heart failure, angina requiring a stent, angioplasty or hospitalisation, myocardial infarction and stroke. We also recorded the use of lipid lowering drugs (LLD). Finally, we tested cognitive performance with the Mini-Mental State Examination (MMSE; cognitive impairment was defined as a score <24)^2^, and the presence of depressive symptoms with the 30-item Geriatric Depression Scale (GDS; a score >10 indicating probable depression)^3^.

***Biochemical data.*** Venous blood samples were collected from participants at baseline after an overnight fast, then centrifuged and stored at −80 °C. For the purposes of our study, in addition to assess LDL-cholesterol, HbA1c and fasting glycaemia, we examined serum creatinine, fibrinogen levels and the erythrocyte sedimentation rate (ESR). Routine biochemical analyses were performed at the local hospital following standard quality-control procedures. Serum creatinine was measured by the standard creatinine Jaffe method (Roche Diagnostics, Germany) and used to compute the glomerular filtration rate (GFR, ml/min) using the Modification of Diet in Renal Disease Study formula. ESR was measured in sodium citrate–anticoagulated blood samples by the Westergren method.

**REFERENCES**

1. Pickering TG, Hall JE, Appel LJ, et al. Recommendations for blood pressure measurement in humans and experimental animals: part 1: blood pressure measurement in humans: a statement for professionals from the Subcommittee of Professional and Public Education of the American Heart Association Cou. *Circulation*. 2005;111(5):697-716. doi:10.1161/01.CIR.0000154900.76284.F6

2. Tombaugh TN, McIntyre NJ. The mini-mental state examination: a comprehensive review. *J Am Geriatr Soc*. 1992;40(9):922-935. http://www.ncbi.nlm.nih.gov/pubmed/1512391. Accessed December 8, 2015.

3. Yesavage JA, Brink TL, Rose TL, et al. Development and validation of a geriatric depression screening scale: a preliminary report. *J Psychiatr Res*. 1982;17(1):37-49. http://www.ncbi.nlm.nih.gov/pubmed/7183759. Accessed August 25, 2014.

**Figure A1. Frequency of healthy cardiovascular factors in the men (n=1187) and women (n=1708) included in our sample**

**
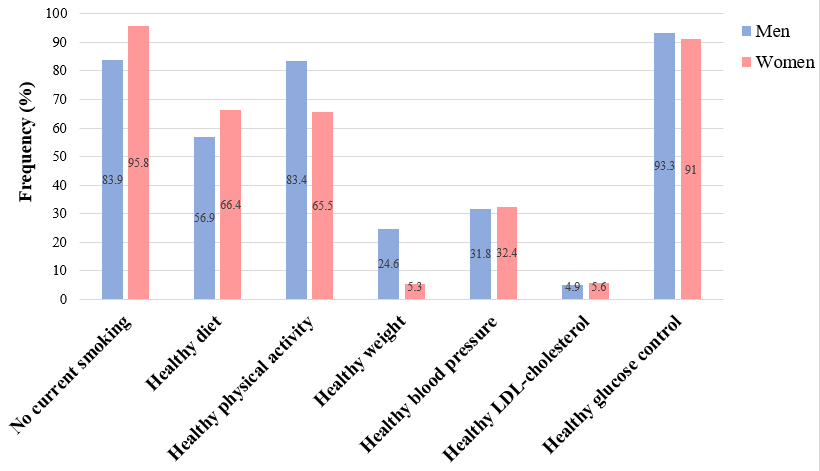
**

**Table A1. Cox regression on the associations between cardiovascular risk profile with 20-year all-cause and cardiovascular mortality in older men and women, stratified by age class**

|  | **Hazard ratios and 95% Confidence Intervals for mortality** | | | | | |
| --- | --- | --- | --- | --- | --- | --- |
|  | **65-74 years** | | **75-84 years** | | **≥85** | |
|  | **Men** | **Women** | **Men** | **Women** | **Men** | **Women** |
|  | **All-cause mortality** | | | | | |
| **Cardiovascular risk profile** | |  |  |  |  |  |
| **Very high** | 1.00 | 1.00 | 1.00 | 1.00 | 1.00 | 1.00 |
| **High** | 0.82 (0.61-1.12) | 0.61 (0.44-0.84)** | 0.76 (0.49-1.18) | 0.72 (0.56-0.93)* | 0.92 (0.53-1.60) | 0.92 (0.65-1.32) |
| **Medium** | 0.61 (0.45-0.83)** | 0.54 (0.40-0.74)*** | 0.76 (0.50-1.15) | 0.64 (0.50-0.82)*** | 0.70 (0.42-1.18) | 0.92 (0.62-1.34) |
| **Low** | 0.52 (0.36-0.74)*** | 0.51 (0.36-0.73)*** | 0.77 (0.48-1.23) | 0.58 (0.43-0.80)*** | 0.77 (0.44-1.32) | 0.93 (0.55-1.57) |
| **Very low** | 0.35 (0.18-0.69)*** | 0.36 (0.18-0.74)*** | 0.98 (0.52-1.84) | 0.41 (0.18-0.94)* | 0.67 (0.31-1.48) | 0.22 (0.05-0.96)* |
| ***p per trend*** | <0.001 | <0.001 | 0.88 | <0.001 | 0.1713 | 0.2304 |
|  | **Cardiovascular mortality** | | | | | |
| **Cardiovascular risk profile** |  |  |  |  |  |  |
| **Very high** | 1.00 | 1.00 | 1.00 | 1.00 | 1.00 | 1.00 |
| **High** | 0.63 (0.38-1.06) | 0.61 (0.38-1.00)* | 1.03 (0.50-2.15) | 0.67 (0.48-0.93)* | 1.09 (0.52-2.27) | 0.90 (0.57-1.41) |
| **Medium** | 0.66 (0.40-1.08) | 0.56 (0.35-0.90)* | 1.11 (0.55-2.24) | 0.53 (0.38-0.75)*** | 0.86 (0.43-1.72) | 0.83 (0.50-1.37) |
| **Low** | 0.36 (0.19-0.68)** | 0.49 (0.28-0.86)* | 1.16 (0.54-2.51) | 0.52 (0.35-0.80)** | 0.87 (0.42-1.80) | 0.78 (0.38-1.55) |
| **Very low** | 0.19 (0.04-0.80)* | 0.20 (0.05-0.87)* | 1.48 (0.56-3.91) | 0.12 (0.02-0.87)* | 0.30 (0.08-1.16) | 0.19 (0.02-1.51) |
| ***p per trend*** | 0.001 | 0.008 | 0.3825 | <0.001 | 0.1171 | 0.1558 |

Model adjusted for educational level (≥5 vs <5 years), monthly income (< vs ≥500€), living arrangements (living with somebody vs living alone), chronic obstructive pulmonary disease, osteoarticular diseases, cancer, cognitive impairment, cardiovascular diseases. *p<0.05; **p<0.01; ***p<0.001

**Table A2. Association between cardiovascular risk factors with 10- and 20-year all-cause and cardiovascular mortality**

|  | **Hazard ratios and 95% Confidence Intervals for mortality** | | | |
| --- | --- | --- | --- | --- |
|  | **All-cause** | | **Cardiovascular** | |
|  | **Men** | **Women** | **Men** | **Women** |
| **Cardiovascular risk profile** | **10-year follow-up** | | | |
| **Healthy glucose control (yes vs no)** | 0.79 (0.59-1.06) | 0.60 (0.48-0.75)*** | 0.94 (0.60-1.47) | 0.80 (0.57-1.13) |
| **Healthy LDL-cholesterol (yes vs no)** | 1.11 (0.76-1.62) | 1.36 (0.95-1.95) | 1.06 (0.59-1.92) | 1.26 (0.73-2.19) |
| **Healthy blood pressure (yes vs no)** | 0.95 (0.80-1.13) | 0.87 (0.73-1.03) | 0.87 (0.68-1.13) | 0.81 (0.63-1.03) |
| **Healthy physical activity (yes vs no)** | 0.67 (0.55-0.81)*** | 0.77 (0.65-0.92)** | 0.63 (0.48-0.83)** | 0.60 (0.47-0.77) |
| **Healthy diet (yes vs no)** | 0.94 (0.80-1.10) | 0.90 (0.76-1.06) | 0.91 (0.72-1.16) | 0.75 (0.60-0.94)* |
| **No current smoking habits (yes vs no)** | 0.75 (0.60-0.93)** | 0.82 (0.50-1.34) | 0.90 (0.63-1.28) | 2.36 (0.75-7.44) |
| **Healthy weight (yes vs no)** | 1.08 (0.90-1.29) | 0.85 (0.57-1.25) | 1.01 (0.77-1.33) | 0.89 (0.53-1.51) |
|  |  |  |  |  |
|  | **20-year follow-up** | | | |
| **Healthy glucose control (yes vs no)** | 0.74 (0.59-0.94)* | 0.63 (0.53-0.75)*** | 0.92 (0.63-1.36) | 0.83 (0.63-1.10) |
| **Healthy LDL-cholesterol (yes vs no)** | 0.99 (0.73-1.34) | 1.43 (1.12-1.82)** | 1.00 (0.63-1.59) | 1.18 (0.80-1.73) |
| **Healthy blood pressure (yes vs no)** | 0.91 (0.80-1.04) | 0.90 (0.80-1.01) | 0.84 (0.68-1.04) | 0.81 (0.68-0.96)* |
| **Healthy physical activity (yes vs no)** | 0.72 (0.60-0.85)*** | 0.82 (0.73-0.92)** | 0.66 (0.52-0.85)** | 0.70 (0.60-0.83)*** |
| **Healthy diet (yes vs no)** | 0.90 (0.79-1.02) | 0.92 (0.82-1.03) | 0.97 (0.80-1.18) | 0.87 (0.74-1.02) |
| **No current smoking habits (yes vs no)** | 0.75 (0.63-0.89)*** | 0.66 (0.50-0.87)** | 0.97 (0.73-1.29) | 1.10 (0.66-1.82) |
| **Healthy weight (yes vs no)** | 1.05 (0.91-1.22) | 0.92 (0.71-1.18) | 1.04 (0.84-1.30) | 1.02 (0.72-1.44) |

Model adjusted for educational level (≥5 vs <5 years), monthly income (< vs ≥500€), living arrangements (living with somebody vs living alone), chronic obstructive pulmonary disease, osteoarticular diseases, cancer, cognitive impairment, cardiovascular diseases. *p<0.05; **p<0.01; ***p<0.001
